# Bifunctional covalent organic framework as a heterogeneous acid photocatalyst for solvent-free microwave-assisted synthesis of isobornyl methacrylate

**DOI:** 10.1039/d6ra06931b

**Published:** 2026-09-29

**Authors:** Quang Tam Huynh, Yu-Fang Lin, Quoc Thanh Vo, Ken-Lin Chang

**Affiliations:** a Institute of Fundamental and Applied Sciences, Duy Tan University Ho Chi Minh City 70000 Vietnam huynhquangtam@dtu.edu.vn; b Faculty of Environment and Chemical Engineering, Duy Tan University Da Nang 50000 Vietnam; c Institute of Environmental Engineering, National Sun Yat-Sen University Kaohsiung 804 Taiwan; d College of Environment and Natural Resources, Can Tho University Can Tho City Vietnam; e Net Zero Emissions and Resource Recycling Technology Research Center, National Sun Yat-Sen University Kaohsiung 804 Taiwan; f Department of Public Health, College of Health Sciences, Kaohsiung Medical University Kaohsiung 807 Taiwan; g Center for Emerging Contaminants Research, National Sun Yat-Sen University Kaohsiung 804 Taiwan

## Abstract

Isobornyl methacrylate (IBOMA) is a bio-based specialty monomer with broad applications in coatings, adhesives, and dental materials. However, its conventional synthesis *via* the acid-catalyzed esterification of camphene with methacrylic acid suffers from poor catalyst recyclability, corrosive waste generation, and low energy efficiency. This study reports the first application of a sulfonated covalent organic framework, TpPa–SO_3_H, as a bifunctional heterogeneous catalyst for solvent-free IBOMA synthesis under combined visible-light irradiation and microwave heating. TpPa–SO_3_H was synthesized *via* both solvothermal and microwave-assisted routes, yielding chemically identical frameworks, with the microwave variant exhibiting a 45% greater total pore volume at 0.518 *vs.* 0.357 cm^3^ g^−1^. Under conditions optimized by the Box–Behnken response surface methodology using 5 wt% catalyst at 80 °C, 60 min, and a camphene-to-methacrylic acid molar ratio of 1 : 3, a maximum IBOMA yield of 90.97% was achieved. The catalyst retained above 80% yield over five reuse cycles, and *E*-factor analysis demonstrated 38- to 76-fold lower waste generation than with H_2_SO_4_, *p*-TsOH, and betaine hydrochloride. An environmental impact assessment further showed that the microwave-synthesized catalyst achieved a global warming potential of 0.0265 kg CO_2_ eq per kg IBOMA, which was 18–71% lower than that of three homogeneous acids, establishing TpPa–SO_3_H as an efficient and environmentally favorable catalyst for bio-based monomer synthesis.

## Abstract 2

A recyclable catalyst enables the efficient synthesis of bio-based isobornyl methacrylate (IBOMA), offering a sustainable route to this specialty monomer for coatings, adhesives, and dental materials.

## Graphical abstract

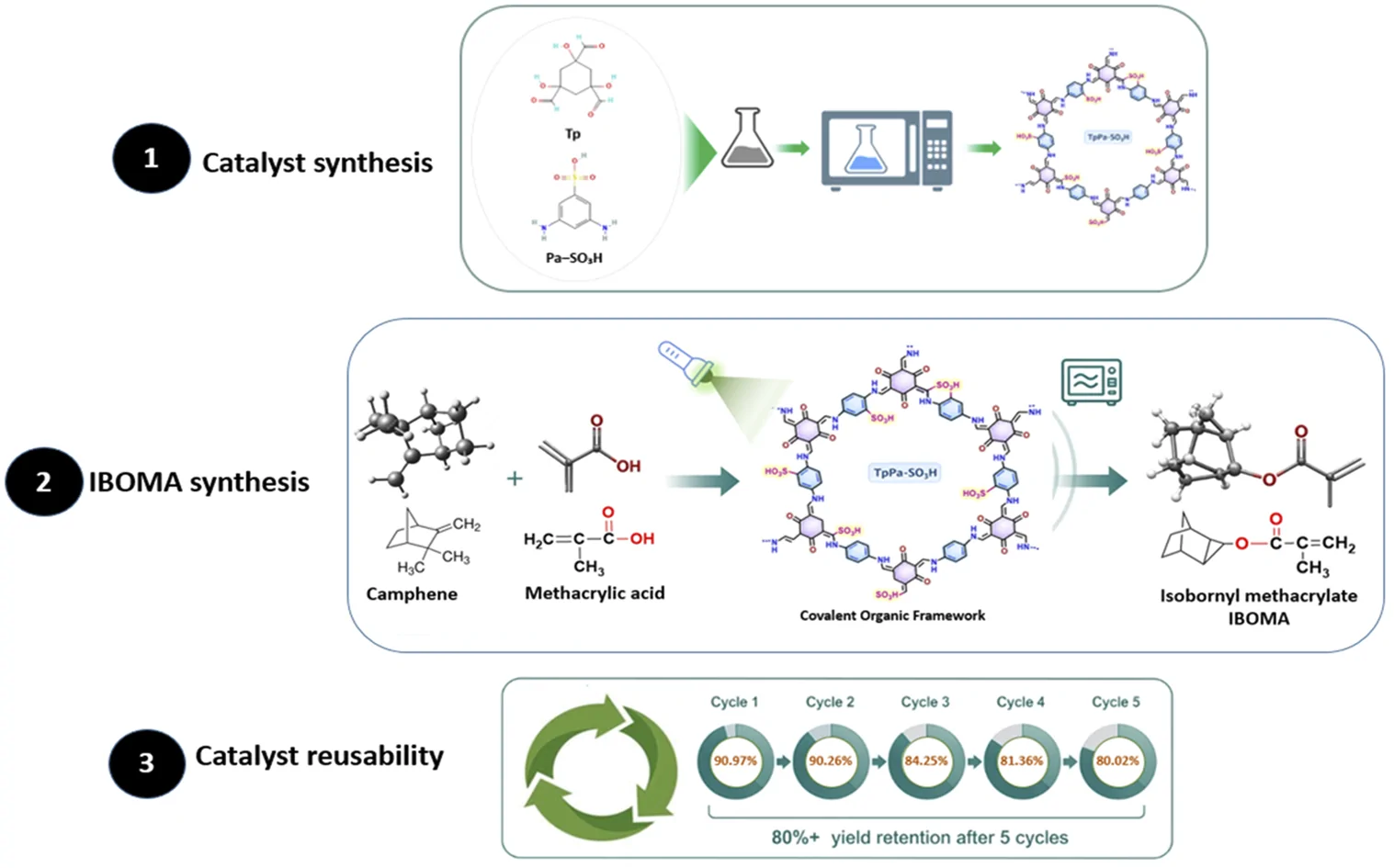

## 1. Introduction

Isobornyl methacrylate (IBOMA) is a bio-based specialty monomer widely used in UV-curable coatings, adhesives, printing inks, dental materials, and drug-delivery systems.^1,2^ Its rigid bicyclic structure imparts an exceptional combination of high glass transition temperature, excellent weatherability, low water absorption, strong adhesion, and low polymerisation shrinkage properties that are difficult to achieve simultaneously with conventional acrylic monomers.^3^ From a sustainability perspective, IBOMA is derived from camphene,^4^ which is obtained from α-pinene, a renewable constituent of pine turpentine, making it an attractive bio-based alternative to petroleum-derived specialty monomers.

The industrial production of IBOMA traditionally relies on the acid-catalyzed esterification of camphene with methacrylic acid *via* a Wagner–Meerwein rearrangement mechanism.^5^ However, conventional catalysts, such as sulfuric acid and strong-acid ion-exchange resins, suffer from significant drawbacks, including equipment corrosion, waste generation, and catalyst deactivation.^6^ Although various heterogeneous acid catalysts have been developed for this kind of esterification process, they generally require prolonged heating and present challenges related to cost, selectivity, and catalyst stability.^6–8^ Consequently, the development of a recyclable and energy-efficient catalytic system for IBOMA synthesis remains an important challenge.

Guided by the principles of green chemistry, photocatalysis offers a promising strategy for developing a more sustainable biochemical synthesis process by utilizing light energy instead of conventional thermal heating, thereby improving energy efficiency and reducing environmental impacts.^9^ Among visible-light-responsive photocatalysts, covalent organic frameworks (COFs) have attracted considerable attention because of their crystalline porous structures, high surface areas, metal-free composition, tunable electronic properties, and strong visible-light absorption.^9,10^ In particular, β-ketoenamine-linked COFs exhibit excellent chemical stability under acidic conditions,^11^ making them suitable for acid-catalyzed reactions.

Microwave (MW) irradiation offers an additional opportunity to intensify photocatalytic synthesis. Beyond rapid volumetric dielectric heating that reduces reaction times and energy consumption, MW irradiation exerts a specific non-thermal effect on metal-oxide photocatalysts: the oscillating electromagnetic field generates oxygen vacancy defect sites that suppress electron–hole recombination and increase the photocatalytic quantum yield.^12^ This integration achieves a 2- to 5-fold rate enhancement compared with standalone photocatalysis, providing a practical approach for applications in food sterilizing, polymer synthesizing, organic synthesis, and sewage treatment.^13^

Despite independent advances in COF synthesis,^14,15^ their integration using a sulfonated COF as a bifunctional acid–photocatalyst for terpene-derived monomer synthesis has not been reported. The present work addresses this gap by demonstrating for the first time the MW-assisted visible-light photocatalytic synthesis of IBOMA using TpPa–SO_3_H as the heterogeneous catalyst. The novelty lies in a three-way synergy: the –SO_3_H groups provide Brønsted acidity for the Wagner–Meerwein rearrangement of camphene, visible-light excitation generates reactive charge carriers that drive the esterification pathway, and MW heating accelerates mass and energy transfer throughout the catalyst bed, collectively overcoming the three principal limitations of existing IBOMA processes: catalyst fouling, long reaction times, and dependence on corrosive liquid acids.

## 2. Materials and methodology

### 2.1. Reagents and biomass

All chemicals used in this study were commercially available and did not undergo additional purification. 1,3,5-Triformylphloroglucinol (C_9_H_6_O_6_, ≥95%), 2,5-diaminobenzenesulfonic acid (C_6_H_8_N_2_O_3_S, 98%), and anhydrous 1,4-dioxane (C_4_H_8_O_2_, 99%) were purchased from Alfa Aesar (Massachusetts, US). Amberlyst-15 (dry form), acetone (C_3_H_6_O, 99%), and ethanol (C_2_H_6_O, 99%) were purchased from Sigma-Aldrich (Darmstadt, Germany). *N*,*N*-Dimethylformamide (DMF, C_3_H_7_NO, 99%), camphene (C_10_H_16_, 75%), and methacrylic acid (MAA, C_4_H_6_O_2_, 98%) were purchased from Merck KGaA (Darmstadt, Germany).

### 2.2. Catalyst synthesis

TpPa–SO_3_H COF was synthesized *via* a solvothermal condensation reaction between 1,3,5-triformylphloroglucinol (Tp) and 2,5-diaminobenzenesulfonic acid (Pa–SO_3_H).^16,17^ Briefly, Tp (63 mg, 0.30 mmol) and Pa–SO_3_H (94 mg, 0.50 mmol) were dissolved in 2.0 mL of 1,4-dioxane under sonication for 5 min. Subsequently, 0.2 mL of 3 M acetic acid was added as the catalyst, and the mixture was stirred gently for 1 min to obtain a homogeneous pale-yellow solution. The resulting solution was transferred into a Teflon-lined stainless-steel autoclave, which was sealed and purged three times with nitrogen gas to establish an inert atmosphere. The autoclave was heated to 120 °C at a ramp rate of 2 °C min^−1^ and maintained at this temperature for 72 h. After naturally cooling to room temperature, the resulting deep red-brown precipitate was collected by vacuum filtration through a 0.22 µm PTFE membrane. The solid product was washed sequentially with *N*,*N*-dimethylformamide (3 × 10 mL), ethanol (3 × 10 mL), and acetone (1 × 10 mL) to remove residual monomers and impurities. Finally, the material was dried under vacuum at 60 °C for 12 h and stored in a sealed vial under a nitrogen atmosphere until further use.

TpPa–SO_3_H was synthesized *via* a microwave-assisted solvothermal condensation.^18^ Tp (63 mg, 0.30 mmol) and Pa–SO_3_H (94 mg, 0.50 mmol) were dispersed in anhydrous 1,4-dioxane (2.0 mL) and 3 M acetic acid (0.2 mL) in a 10 mL sealed microwave vessel and sonicated for 5 min. The mixture was heated to 120 °C within 5 min and maintained at 120 °C for 60 min with a maximum power of 300 W. After cooling to below 40 °C, the precipitated solid was isolated by vacuum filtration, washed sequentially with *N*,*N*-dimethylformamide (3 × 10 mL), ethanol (3 × 10 mL), and acetone (1 × 10 mL) to remove residual monomers and impurities. Finally, the material was dried under vacuum at 60 °C for 12 h and stored in a sealed vial under a nitrogen atmosphere until further use.

### 2.3. Catalyst characterization

The functional groups of the catalyst were characterized by Fourier-transform infrared (FTIR) spectroscopy using a PerkinElmer Spectrum instrument (PerkinElmer, USA). The specific surface area was determined using the Brunauer–Emmett–Teller (BET) method with a 3Flex analyzer (Micromeritics Instrument Corporation, USA). The total pore volume and average pore size were calculated using the Barrett–Joyner–Halenda (BJH) method. The thermal stability of the catalyst was analyzed by thermogravimetric analysis (TGA) using a TGA instrument (Mettler Toledo, Switzerland) over a temperature range of 30–800 °C. The morphology of the catalyst was examined using scanning electron microscopy (SEM) with a JSM-6490LV microscope (JEOL, Japan). The SEM analysis was conducted under high vacuum at an accelerating voltage of 20 kV in secondary electron mode. Solid-state UV-vis absorption spectra were recorded using an Evolution Pro UV-vis spectrophotometer (Thermo Fisher Scientific, USA).

### 2.4. Isobornyl methacrylate synthesis

IBOMA synthesis was conducted under solvent-free conditions using a camphene-to-methacrylic acid molar ratio ranging from 1 : 1 to 1 : 1.5 and a TpPa–SO_3_H catalyst loading of 5–10 wt%. The reactions were conducted at 50–80 °C under 450 nm visible-light irradiation using a 300 W microwave reactor, with reaction times of 15–30 min. Ambient air can be used as the reaction atmosphere, while 0.05 wt% phenothiazine is added to suppress methacrylic acid polymerization. Gas chromatography with flame ionization detection (GC–FID) was employed for the analysis of IBOMA. Prior to analysis, the reaction samples were diluted with tetrahydrofuran (THF) and filtered through a 0.22 µm syringe filter. Separation was carried out on a DB-5 capillary column (30 m × 0.25 mm, 0.25 µm film thickness). Helium was used as the carrier gas at a constant flow rate of 1.0 mL min^−1^. Samples were injected in split mode with a split ratio of 20 : 1, and the injector temperature was maintained at 250 °C. The FID detector temperature was set at 280 °C.

For the esterification of camphene with MAA to produce IBOMA, the yield is calculated using eqn (1) based on the limiting reactant, which is camphene. The yield of products (mol%) is calculated as follows:1
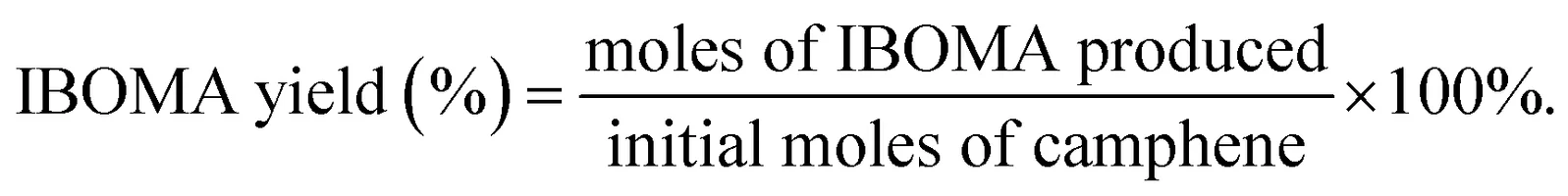


## 3. Results and discussion

### 3.1. Catalyst characterization

A comparative study of TpPa–SO_3_H synthesized *via* solvothermal and microwave-assisted methods showed that both approaches produced materials with similar chemical and thermal properties, as presented in Fig. 1.

**Fig. 1:**
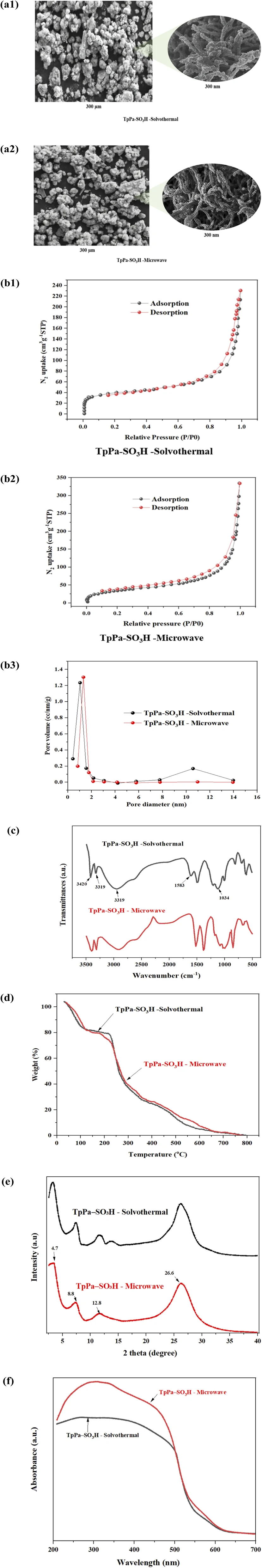
Characterization of TpPa–SO_3_H synthesized by solvothermal and microwave-assisted methods. (a) SEM images under low and high magnifications of TpPa–SO_3_H synthesized by (a1) solvothermal and (a2) microwave-assisted methods. (b) N_2_ adsorption–desorption isotherms of TpPa–SO_3_H synthesized by (b1) solvothermal and (b2) microwave-assisted methods and (b3) pore size distributions. (c) FTIR spectra, (d) TGA profiles, (e) XRD spectra, and (f) UV-vis absorption spectra.

The morphologies of TpPa–SO_3_H prepared by solvothermal and microwave-assisted methods are shown in Fig. 1(a). At low magnification (300 µm), both samples displayed similarly irregular, agglomerated particle clusters. At a higher magnification (300 nm), however, clear structural differences emerged: the solvothermal sample showed short, densely packed rod-like bundles with partial aggregation, whereas the microwave sample exhibited elongated, well-defined nanofibers forming an open, loosely interwoven network.

The textural properties of TpPa–SO_3_H synthesized by solvothermal and microwave methods were evaluated, as shown in Fig. 1(b). Both samples exhibited type IV isotherms with hysteresis loops at intermediate to high relative pressures (*P*/*P*_0_ = 0.4–1.0), characteristic of mesoporous materials. The microwave-synthesized sample reached a maximum N_2_ uptake of 334.5 cm^3^ g^−1^ (STP), considerably higher than 230.6 cm^3^ g^−1^ (STP) recorded for the solvothermal counterpart, reflecting its more open pore structure. BET analysis yielded comparable specific surface areas of 122.0 and 126.7 m^2^ g^−1^ for the microwave and solvothermal samples, respectively. In contrast, the total pore volume of TpPa–SO_3_H–microwave at 0.518 cm^3^ g^−1^ was approximately 45% greater than that of TpPa–SO_3_H–solvothermal at 0.357 cm^3^ g^−1^; this difference contributes more directly to catalytic accessibility than the specific surface area alone. Pore size distribution analysis revealed dominant sub-2 nm pore features centered at 1.04 nm for the solvothermal sample and 1.32 nm for the microwave sample. The solvothermal sample additionally exhibited secondary pore contributions at 10.6 and 14.0 nm, likely arising from interparticle voids associated with its more compact, aggregated morphology. The superior pore volume of TpPa–SO_3_H–microwave is consistent with the open, fibrous nanostructure observed by SEM and is expected to enhance reactant diffusion into the framework channels, thereby improving the accessibility of the bifunctional –SO_3_H and photocatalytic active sites during IBOMA synthesis.

The FTIR spectra of both TpPa–SO_3_H samples in Fig. 1(c) confirmed the successful formation of the COF framework and incorporation of sulfonic acid groups. The broad absorption bands at 3420 and 3319 cm^−1^ are attributed to the O–H stretching of–SO_3_H groups and residual adsorbed moisture.^19^ The band at 1583 cm^−1^ corresponds to the C=N stretching of the imine linkage,^20^ confirming the condensation reaction between the aldehyde (Tp) and amine (Pa) building blocks. The characteristic absorption at 1034 cm^−1^ is assigned to the S=O stretching of the sulfonate group,^21^ verifying successful –SO_3_H functionalization within the COF backbone. Both samples display essentially identical spectral features, confirming that the two synthetic routes yield the same chemical framework, with differences residing in morphology and texture rather than composition.

The thermal stability of both samples was evaluated by TGA, as shown in Fig. 1(d). Both TpPa–SO_3_H–solvothermal and TpPa–SO_3_H–microwave exhibited similar three-stage decomposition profiles. The initial weight loss below 150 °C is attributed to the removal of physically adsorbed water and residual solvent trapped within the pore channels. A second, more pronounced weight loss occurring between approximately 200 °C and 350 °C corresponds to the decomposition of –SO_3_H groups and the partial degradation of imine linkages. The major framework decomposition takes place above 350 °C, consistent with the breakdown of the β-ketoenamine backbone of TpPa COF. Both samples retained comparable thermal stability up to 300 °C, confirming that microwave synthesis does not compromise the thermal robustness of the resulting framework. The overall decomposition profile is consistent with previously reported TpPa-based COFs in the literature.

The XRD patterns in Fig. 1(e) exhibited distinct diffraction peaks, confirming the formation of an ordered crystalline structure. A strong reflection at approximately 2*θ* = 4.7° can be assigned to the (100) plane, characteristic of the periodic in-plane structure of TpPa–SO_3_H. Additional reflections at 8.8° and 12.8° further support the ordered framework structure, while the broad peak at approximately 26.6° is associated with the (001) reflection arising from the stacking of COF layers.^22,23^ The similar diffraction features observed for the solvothermal and microwave-synthesized TpPa–SO_3_H indicate that both synthesis routes successfully produced the same crystalline COF framework.

The UV-vis absorption spectra of TpPa–SO_3_H synthesized by the solvothermal and microwave-assisted methods are shown in Fig. 1(f). Both samples exhibited strong absorption in the UV region with an extended absorption tail into the visible-light region, indicating their ability to interact with visible light. The microwave-synthesized TpPa–SO_3_H showed a relatively stronger absorption than the solvothermal sample over the 200–500 nm range, which may contribute to its enhanced light-assisted catalytic performance. However, no specific band-gap or charge-carrier behavior is assigned solely from the absorption spectra.

### 3.2. Catalyst effectiveness

The catalytic performance of TpPa–SO_3_H was evaluated against several conventional Brønsted acid catalysts, including sulfuric acid, betaine hydrochloride, and *p*-toluenesulfonic acid (*p*-TsOH), under both visible-light irradiation and dark conditions, as presented in Fig. 2. The reactions were conducted under solvent-free conditions in a 300 W microwave reactor equipped with a 450 nm visible-light LED source. Camphene and methacrylic acid were introduced into the reactor at a molar ratio of 1 : 1.5, together with 5 wt% TpPa–SO_3_H catalyst and 0.05 wt% phenothiazine as a radical inhibitor to prevent the undesired polymerization of methacrylic acid during the reaction.

**Fig. 2:**
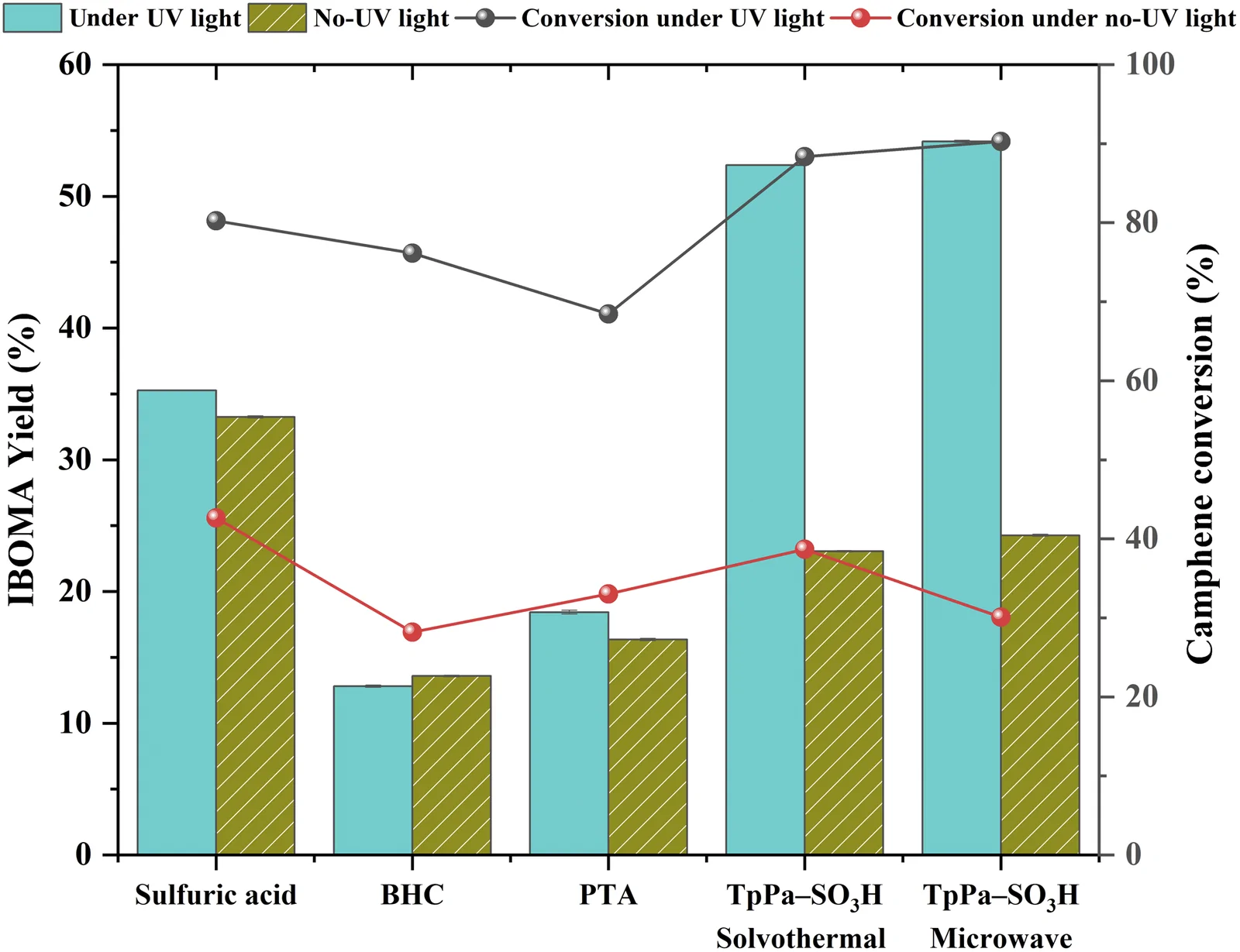
Catalytic performance of different catalysts for IBOMA synthesis under visible-light irradiation and dark conditions.

Among all catalysts tested, TpPa–SO_3_H–microwave delivered the highest IBOMA yield of 54.17% and camphene conversion of 90.27% under visible-light irradiation, followed closely by TpPa–SO_3_H–solvothermal with 52.36% and 88.36%, respectively. In contrast, the conventional homogeneous acids performed significantly worse, with H_2_SO_4_ giving the highest yield among the reference catalysts at only 35.26%, while betaine hydrochloride and *p*-TsOH yielded 12.81% and 18.43%, respectively. The notably large gaps between camphene conversion and IBOMA yield observed for the homogeneous acids, particularly betaine hydrochloride (76.14% conversion and 12.81% yield) and *p*-TsOH (68.45% conversion and 18.43% yield), suggest poor selectivity toward the esterification pathway, with camphene likely undergoing competing rearrangement or oligomerization side reactions in the absence of the structured pore environment provided by the COF framework.

The role of visible-light irradiation was most clearly revealed by comparing yields obtained under visible-light and no-visible-light conditions. For all three conventional acid catalysts, visible-light irradiation produced negligible improvement in the IBOMA yield, confirming that light alone does not accelerate acid-catalyzed esterification in the absence of a photoactive material. In striking contrast, both COF catalysts exhibited a dramatic increase of approximately 30% in IBOMA yield upon visible-light irradiation, rising from around 23–24% to over 52–54%. This unambiguous visible-light-dependent enhancement provides direct evidence for the bifunctional role of TpPa–SO_3_H, in which the Brønsted acid –SO_3_H groups and the visible-light-activated COF framework act cooperatively: the –SO_3_H sites drive the Wagner–Meerwein rearrangement and esterification, while the light-responsive β-ketoenamine framework contributes to the enhanced catalytic activity under visible-light irradiation.^24^ The marginally superior performance of TpPa–SO_3_H–microwave over its solvothermal counterpart is consistent with its higher total pore volume (0.518 *vs.* 0.357 cm^3^ g^−1^), which facilitates greater accessibility of active sites to the bulky camphene substrate within the porous COF structure.

### 3.3. Parameter evaluations

The effects of the camphene-to-methacrylic acid molar ratio, catalyst loading, reaction temperature, and reaction time on the synthesis of IBOMA were systematically investigated by monitoring the IBOMA yield and camphene conversion. The experimental results are presented in Fig. 3.

**Fig. 3:**
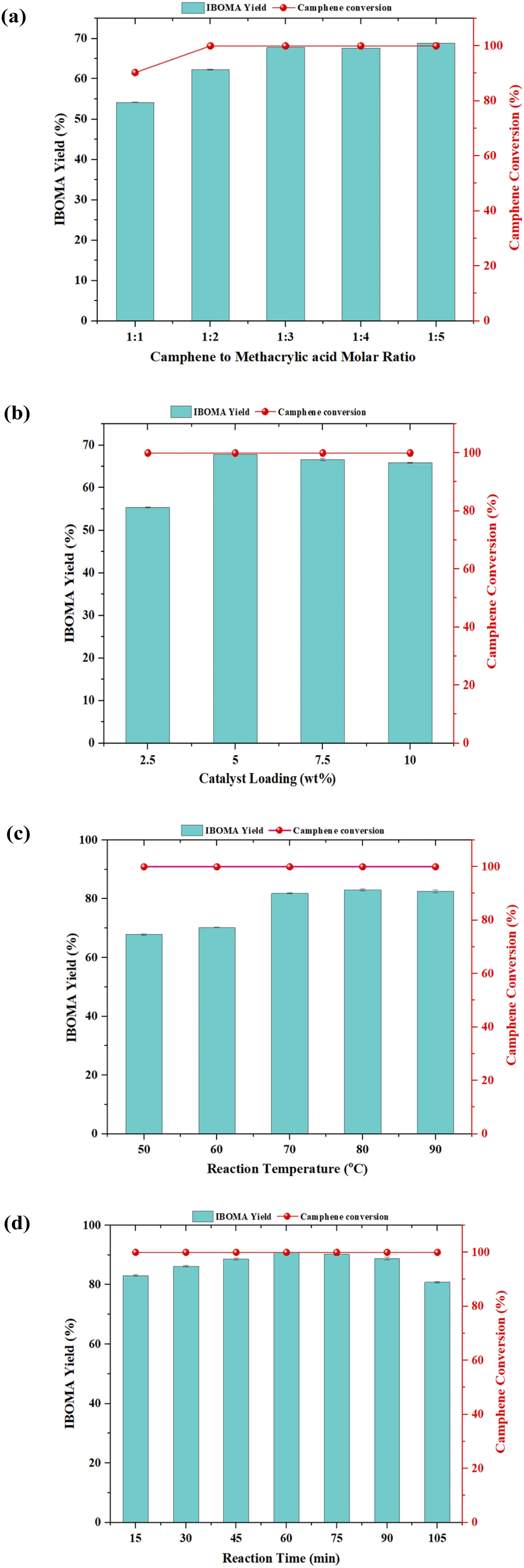
Effect of the reaction parameters on IBOMA yield and camphene conversion: (a) camphene-to-methacrylic acid molar ratio, (b) catalyst loading, (c) reaction temperature, and (d) reaction time.

Among the investigated parameters, the camphene-to-MA molar ratio significantly affected the IBOMA yield. As displayed in Fig. 3(a), increasing the MA ratio from 1 : 1 to 1 : 3 increased the yield from 52.09% to 67.82% by shifting the reaction equilibrium toward ester formation and providing sufficient methacrylic acid for reaction with the carbocation intermediate. Further increasing the ratio to 1 : 4 and 1 : 5 resulted in only slight improvements, indicating that MA was no longer the limiting reactant. Catalyst loading also influenced the reaction selectivity, as presented in Fig. 3(b). Increasing the catalyst amount from 2.5 wt% to 5 wt% enhanced the yield from 55.36% to 67.82% because more Brønsted acid sites were available to promote carbocation formation. However, further increasing the catalyst loading slightly decreased the yield, suggesting that excessive acidity promoted competing reactions such as carbocation rearrangement and methacrylic acid polymerization. A similar trend was observed for reaction temperature and reaction time, as shown in Fig. 3(c) and (d), respectively. Raising the temperature from 50 °C to 80 °C significantly improved the yield by accelerating esterification kinetics from 67.82% to 83.06%, whereas a slight decrease in the yield at 90 °C indicated the occurrence of thermal side reactions. Likewise, extending the reaction time from 15 to 60 min progressively increased the yield to 90.97%, while longer reaction times led to a gradual decline due to secondary reactions, including reversible esterification, polymerization, and possible product degradation. The results of reaction times are displayed in Fig. 3(d). Notably, camphene conversion reached 100% under nearly all conditions, whereas the IBOMA yield remained lower, indicating that a fraction of the converted camphene was consumed in side reactions rather than forming the desired ester.

Overall, the optimization results demonstrate that the highest IBOMA yield of 90.97% was achieved using a camphene-to-MA molar ratio of 1 : 3, a catalyst loading of 5 wt%, a reaction temperature of 80 °C, and a reaction time of 60 min. These conditions provide an optimal balance between reaction kinetics and product selectivity while minimizing the formation of by-products and were therefore selected for subsequent experiments.

### 3.4. The interaction of parameters in IBOMA synthesis effectiveness

While initial screening identified a high-performing condition, this interaction analysis was essential to characterize the process robustness and sensitivity of the reaction. To quantify these synergistic and antagonistic effects, we employed response surface methodology (RSM) *via* a Box–Behnken design in Design-Expert. The experimental design and parameter variations are summarized in Table 1. Eqn (2) was used to make predictions about the response. Multiple regression analysis was then applied to generate a second-order polynomial model describing the relationship between the coded variables and the IBOMA yield as follows:

2 Yield = 81.61 + 9.97 × *A* − 1.07 × *B* + 0.72 × *C* + 2.46 × *AB* − 0.043 × *AC* + 1.07 × *BC* − 17.47 × *A*^2^ − 1.65 × *B*^2^ − 2.20 × *C*^2^,

where yield is the IBOMA yield, and *A*, *B*, and *C* are the coded factors for the test variables: catalyst loading, temperature, and time, respectively.

**Table 1:** Experimental factors and levels used in the Box–Behnken design

Independent variable unit	Unit	Code	Level		
			−1	0	1
Catalyst loading	wt%	*A*	2.5	6.25	10
Reaction temperature	°C	*B*	50	70	90
Reaction time	min	*C*	15	60	105

The response surface plots in Fig. 4 illustrate the interaction effects of catalyst loading, reaction temperature, and reaction time on the IBOMA yield. Unlike one-factor-at-a-time optimization, the response surface methodology (RSM) evaluates the combined influence of reaction variables, providing a more comprehensive understanding of the catalytic process. The curved response surfaces and elliptical contour plots indicate significant interactions among the variables, suggesting that the reaction performance depends on their combined effects rather than individual factors.

**Fig. 4:**
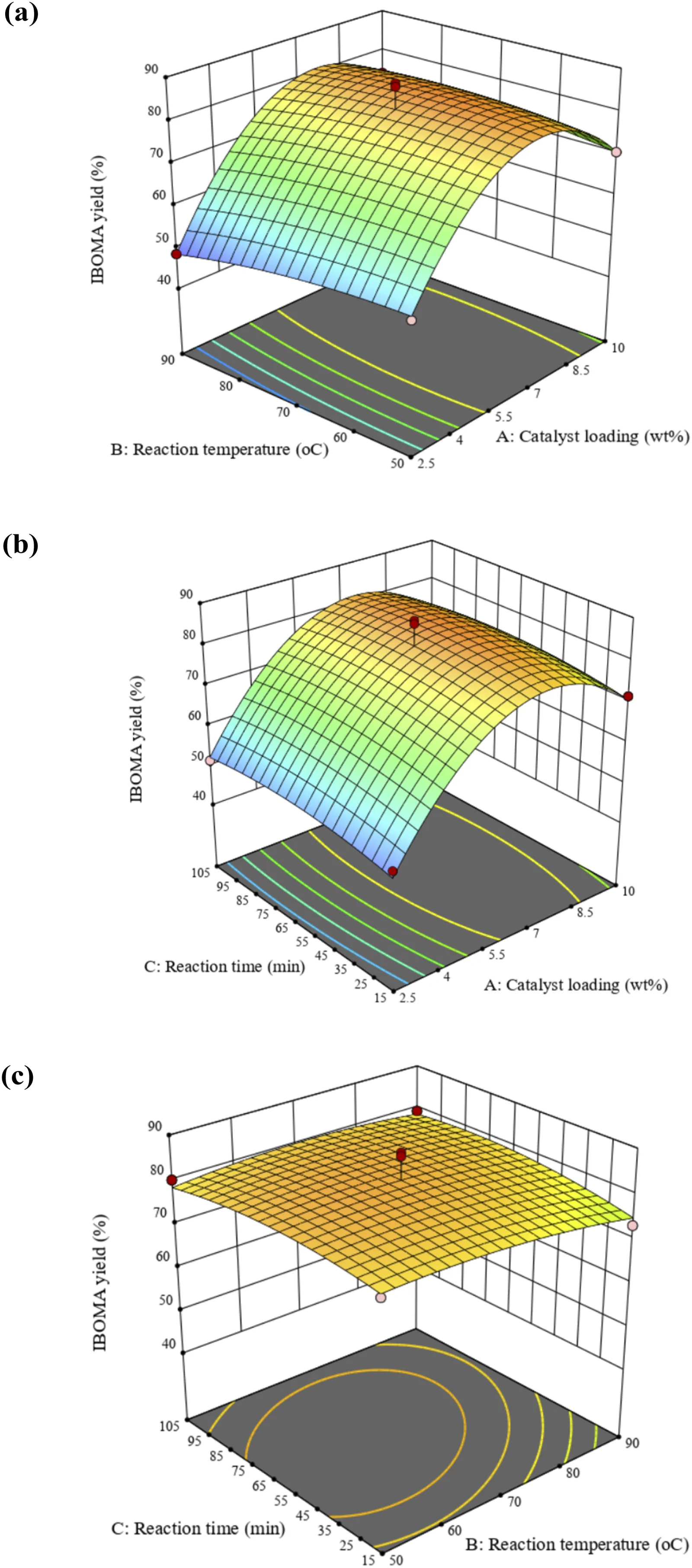
Response surface plots illustrating the interaction effects of the reaction variables on IBOMA yield: (a) catalyst loading *vs.* reaction temperature. (b) Catalyst loading *vs.* reaction time, and (c) reaction temperature *vs.* reaction time.

As shown in Fig. 4(a) and (b), increasing the catalyst loading from 2.5 to approximately 5 wt% significantly improved the IBOMA yield, while further increases produced little improvement or a slight decline, likely due to enhanced side reactions under excessive acidity. Similarly, increasing the reaction temperature from 50 °C to around 80 °C and extending the reaction time to approximately 60 min increased the yield by accelerating esterification kinetics. However, higher temperatures or prolonged reaction times slightly reduced the yield, indicating the occurrence of secondary reactions such as methacrylic acid polymerization and product degradation. The elliptical contour plots further confirm that interactions among catalyst loading, temperature, and reaction time contribute significantly to the reaction performance.

Overall, the response surface analysis demonstrates that the highest IBOMA yield is achieved through a synergistic combination of reaction parameters rather than maximizing a single factor. The optimum region was identified at approximately 5 wt% catalyst loading, 80 °C reaction temperature, and 60 min reaction time, which agrees well with the results obtained from the individual parameter optimization. These findings confirm the reliability of the optimized reaction conditions for efficient IBOMA synthesis.

### 3.5. Proposed mechanism

Fig. 5 shows the proposed reaction mechanism for the synthesis of IBOMA catalyzed by TpPa–SO_3_H. The reaction proceeds through four consecutive steps.

**Fig. 5:**
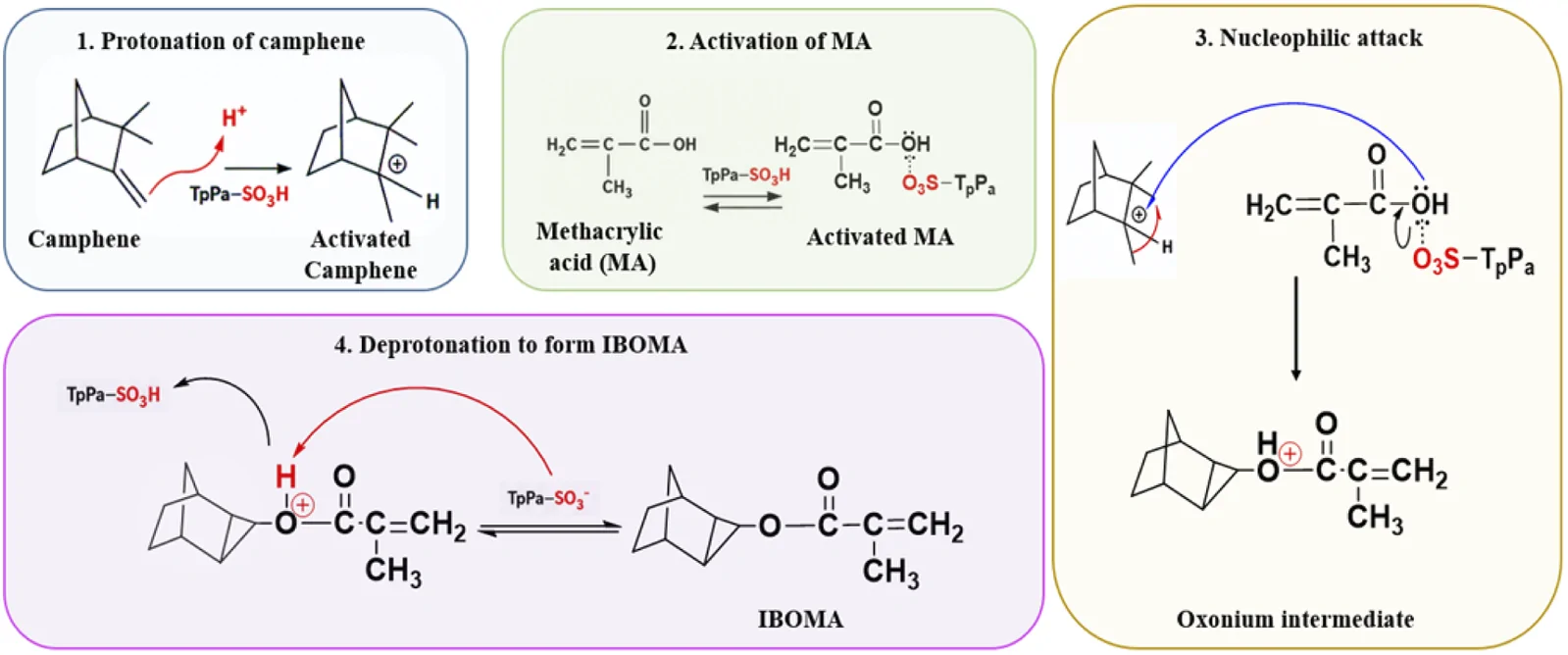
Proposed reaction mechanism for the synthesis of IBOMA catalyzed by TpPa–SO_3_H.

Initially, the Brønsted acidic sulfonic acid (–SO_3_H) groups protonate the double bond of camphene, generating a protonated intermediate that undergoes a Wagner–Meerwein rearrangement to form the stable isobornyl carbocation.^25^ Simultaneously, MA is activated through protonation of its carbonyl oxygen, increasing its electrophilicity. Under visible-light irradiation, the β-ketoenamine framework of TpPa–SO_3_H absorbs light, which contributes to the observed light-assisted catalytic activity.^26^ The activated MA subsequently reacts with the isobornyl carbocation to form an oxonium intermediate, which is stabilized by the sulfonate groups of the catalyst. Finally, deprotonation of the oxonium intermediate yields IBOMA and regenerates the Brønsted acid sites, completing the catalytic cycle. In addition, microwave irradiation provides rapid volumetric heating that reduces reaction time and enhances mass transfer through the porous framework, facilitating reactant diffusion and increasing collision frequency at active sites.^27^

### 3.6. Catalyst reusability

To evaluate the chemical stability and reusability of the TpPa–SO_3_H catalyst, X-ray photoelectron spectroscopy (XPS) was performed before and after five consecutive catalytic cycles, and the catalytic performance was assessed through recycling experiments. The S 2p high-resolution spectra were selected because the sulfur species originate from the catalytically active sulfonic acid (–SO_3_H) groups, making them a sensitive indicator of catalyst stability during the reaction.

As shown in Fig. 6(a) and (b), the S 2p spectra before and after five reaction cycles exhibited nearly identical peak profiles and binding energies, indicating that the chemical state of sulfur remained essentially unchanged after repeated use. The characteristic peaks assigned to sulfonate (–SO_3_^−^) and sulfonic acid (–SO_3_H) species were well preserved, suggesting that the Brønsted acid sites were retained without significant leaching or chemical degradation. Furthermore, the recycling results in Fig. 6(c) showed that the IBOMA yield remained above 80% after five consecutive runs, with only a slight decrease compared with that of the fresh catalyst. The minor loss in catalytic activity is likely attributed to the adsorption of reaction intermediates or products on the catalyst surface and to slight pore blockage rather than to structural decomposition of the catalyst.

**Fig. 6:**
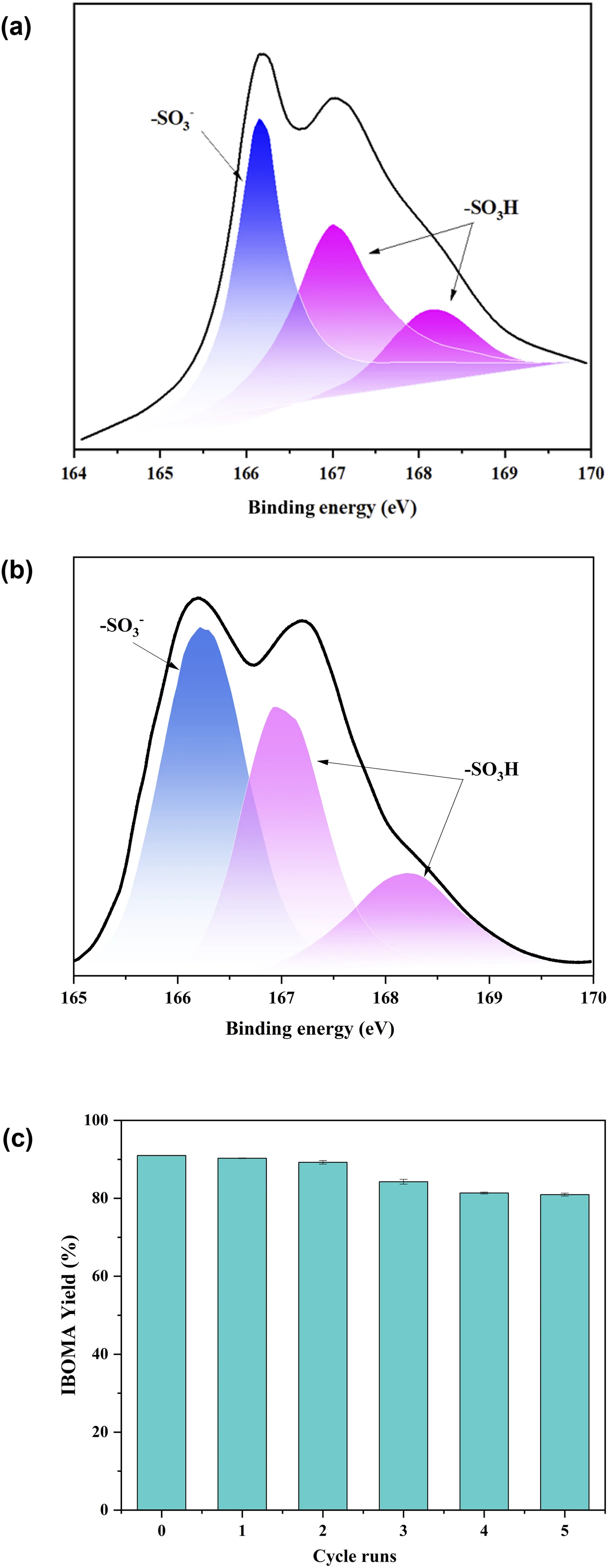
Reusability of the TpPa–SO_3_H catalyst: high-resolution S 2p XPS spectra of the (a) fresh and (b) spent catalyst after five reaction cycles and (c) IBOMA yield over five consecutive recycling runs.

Overall, the consistent S 2p XPS spectra together with the high catalytic activity after repeated use demonstrate the excellent chemical stability and recyclability of the TpPa–SO_3_H catalyst. These results confirm that the sulfonic acid functional groups remain intact during the esterification reaction, enabling sustained catalytic performance and highlighting the potential of TpPa–SO_3_H as a durable heterogeneous catalyst for IBOMA synthesis.

### 3.7. Green chemistry assessment

The environmental efficiency of the TpPa–SO_3_H-catalyzed IBOMA synthesis was quantified using the *E*-factor metric (kg waste per kg product), a widely adopted green chemistry indicator introduced by Sheldon,^28^ using DodecaGreen, the Green Chemistry Portal, and the environmental impact (GWP, kg CO_2_ eq per kg product), calculated using SimaPro v9 with the Ecoinvent v3.6 database and the IMPACT 2002+ impact assessment method.^29^ All calculations were performed on a consistent basis of 1 g camphene with a 1 : 1.5 camphene-to-methacrylic acid (MAA) molar ratio and 5 wt% catalyst loading, corresponding to the screening conditions reported in Section 3.2, to ensure a fair comparison across all catalyst systems. Both include two accounting boundaries: reaction-only, covering solely the IBOMA synthesis step, and integrated, which additionally incorporates the waste and impact generated during catalyst synthesis, amortized over five reuse cycles, consistent with Section 3.6. The results of climate impact were benchmarked against three homogeneous acid catalysts commonly used for this transformation: H_2_SO_4_, *p*-TsOH, and betaine HCl.

Considering only the IBOMA synthesis step, the TpPa–SO_3_H–microwave catalyst exhibited the lowest *E*-factor (0.69), followed by TpPa–SO_3_H–solvothermal (0.72). These values are around 38-, 53-, and 76-fold lower than those of H_2_SO_4_ (27.74), *p*-TsOH (36.70), and betaine hydrochloride (52.63), respectively, as shown in Table 2. The outstanding environmental performance of the COF catalyst is attributed to its heterogeneous and reusable nature, which eliminates the neutralization step and the associated generation of inorganic salts and contaminated wash water. In addition, solvent-free reaction conditions prevent solvent waste during IBOMA synthesis, while the catalyst is recovered by simple filtration and reused, without contributing to the waste inventory. The major waste source is the excess methacrylic acid (0.61 g), which could potentially be recovered by distillation, further reducing the reaction *E*-factor.

**Table 2:** Input data for the *E*-factor calculation of IBOMA synthesis using different catalyst systems (MW = microwave-synthesized and ST = solvothermal)

Chemical/stream	TpPa–SO_3_H MW (g)	TpPa–SO_3_H ST (g)	H_2_SO_4_ (g)	*p*-TsOH (g)	Betaine HCl (g)
**Reactant**					
Camphene	1.0000	1.0000	1.0000	1.0000	1.0000
Methacrylic acid	0.9479	0.9479	0.9479	0.9479	0.9479
Catalyst	0.0974	0.0974	0.0974	0.0974	0.0974
Phenothiazine	0.0005	0.0005	0.0005	0.0005	0.0005
NaOH (neutralization)	—	—	0.0794	0.0226	0.0254

**Product**					
IBOMA	0.8840	0.8545	0.5754	0.3008	0.2091
Water	0.0716	0.0692	0.0466	0.0244	0.0169

**Reaction waste**					
Unreacted methacrylic acid	0.6056	0.6170	0.7251	0.8315	0.8670
Catalyst residue	—	—	0.0974	0.0974	0.0974
Neutralization salts	—	—	0.1410	0.1098	0.0371
Wash water	—	—	15.0000	10.0000	10.0000
**Total reaction waste**	**0.6056**	**0.6170**	**15.9640**	**11.0391**	**11.0019**
**Reaction *E*-factor**	**0.69**	**0.72**	**27.74**	**36.70**	**52.63**

**Catalyst synthesis (allocated over five reuse cycles)**					
Dioxane	0.41	0.41	—	—	—
Acetic acid	0.04	0.04	—	—	—
DMF washing	5.66	5.66	—	—	—
Ethanol washing	4.73	4.73	—	—	—
Acetone washing	1.58	1.58	—	—	—
Total catalyst synthesis waste	**12.42**	**12.42**	—	—	—
**Integrated total waste**	**13.03**	**13.04**	**15.9640**	**11.0391**	**11.0019**
**Integrated *E*-factor**	**14.74**	**15.26**	**27.74**	**36.70**	**52.63**

When the environmental burden of COF synthesis is included and distributed over five reuse cycles, the integrated *E*-factor increases to 14.74 for TpPa–SO_3_H–microwave and 15.26 for TpPa–SO_3_H–solvothermal. This increase is mainly attributed to the solvent-intensive washing process during catalyst preparation, with DMF, ethanol, and acetone accounting for approximately 96% of the synthesis-stage waste. Nevertheless, the integrated *E*-factor remains approximately twofold lower than H_2_SO_4_, 2.7-fold lower than *p*-TsOH, and 3.9-fold lower than betaine hydrochloride, demonstrating that the environmental advantage of the COF catalyst is maintained even under full life-cycle accounting.

The TpPa–SO_3_H(MW)-catalyzed route exhibited the lowest GWP among all catalyst systems evaluated (0.0265 kg CO_2_ eq/kg IBOMA), 18% lower than H_2_SO_4_ (0.0322), 59% lower than *p*-TsOH (0.0643), and 71% lower than betaine–HCl (0.0913). This advantage was most pronounced in categories associated with the neutralization step and its wash-water and salt waste streams, including aquatic ecotoxicity, terrestrial ecotoxicity, and aquatic/terrestrial acidification, where TpPa–SO_3_H(MW) reduced impacts by 47–87% relative to the three homogeneous acids. Consistent with the *E*-factor breakdown above, H_2_SO_4_ showed lower impacts than the COF catalyst in categories linked primarily to upstream chemical production, including carcinogens, non-carcinogens, and non-renewable energy demand, a direct consequence of the solvent-intensive catalyst synthesis step using DMF, dioxane, ethanol, and acetone, identified above as the dominant contributor to the integrated *E*-factor (Table 3).

**Table 3:** Environmental impact indicators for IBOMA synthesis using different catalysts

Impact category	Unit	TpPa–SO_3_H (MW)	H_2_SO_4_	*p*-TsOH	Betaine–HCl
Carcinogens	kg C_2_H_3_Cl eq	0.00054	0.00032	0.000706	0.000956
Non-carcinogens	kg C_2_H_3_Cl eq	0.00015	0.00015	0.000368	0.000455
Respiratory inorganics	kg PM2.5 eq	1.16 × 10^−5^	9.64 × 10^−6^	2.48 × 10^−5^	2.96 × 10^−5^
Ionizing radiation	Bq C-14 eq	0.0897	0.0627	0.17	0.204
Ozone layer depletion	kg CFC-11 eq	1.13 × 10^−9^	5.57 × 10^−10^	1.93 × 10^−9^	1.73 × 10^−9^
Respiratory organics	kg C_2_H_4_ eq	1.03 × 10^−5^	7.5 × 10^−6^	1.61 × 10^−5^	2.3 × 10^−5^
Aquatic ecotoxicity	kg TEG water	1.06	0.809	2.09	2.46
Terrestrial ecotoxicity	kg TEG soil	0.269	0.238	0.589	0.72
Terrestrial acidification and eutrophication	kg SO_2_ eq	0.000197	0.00017	0.000391	0.00051
Land occupation	m2org.arable	0.000105	7.98 × 10^−5^	0.000197	0.000913
Aquatic acidification	kg SO_2_ eq	5.96 × 10^−5^	0.00017	0.000128	0.000162
Aquatic eutrophication	kg PO_4_ P-lim	5.08 × 10^−6^	3.36 × 10^−6^	7.63 × 10^−6^	1.03 × 10^−5^
Global warming	kg CO_2_ eq	0.0265	0.0322	0.0643	0.0913
Non-renewable energy	MJ primary	0.325	0.217	0.5	0.665
Mineral extraction	MJ surplus	0.000635	0.00058	0.00129	0.00172

Taken together, the mass-based (*E*-factor) and climate-impact (GWP) assessments converge on the same conclusion: the heterogeneous, recyclable TpPa–SO_3_H catalyst offers a substantial net environmental benefit over conventional homogeneous acid catalysts for IBOMA synthesis, driven principally by elimination of the neutralization step, while both independently identify catalyst-synthesis solvent selection, particularly DMF use, as the primary target for further improvement.

## 4. Conclusion

This study demonstrated the bifunctional catalytic activity of TpPa–SO_3_H COF, synthesized *via* solvothermal and microwave-assisted routes, for solvent-free IBOMA synthesis under combined visible-light irradiation and microwave heating. Although both synthetic routes produced chemically identical frameworks, the microwave-synthesized variant exhibited a 45% greater total pore volume (0.518 *vs.* 0.357 cm^3^ g^−1^), which contributed to its superior catalytic performance. Under optimized conditions (5 wt% catalyst, 80 °C, 60 min, camphene-to-MA ratio of 1 : 3), TpPa–SO_3_H–microwave achieved a maximum IBOMA yield of 90.97%, substantially outperforming conventional Brønsted acid catalysts. The observed visible-light-dependent enhancement of up to 30% supports the proposed cooperative contribution of the –SO_3_H-driven Wagner–Meerwein rearrangement and the light-responsive COF framework. Catalyst reusability tests showed that the IBOMA yield remained above 80% after five cycles, with XPS confirming the presence of intact sulfonic acid sites. Green chemistry assessment further revealed reaction *E*-factors of 0.69 and 0.72 for the microwave and solvothermal variants, respectively, 38- to 76-fold lower than conventional acid catalysts, with the environmental advantage retained under full life-cycle accounting. The environmental impact assessment further showed that the microwave-synthesized catalyst achieved the lowest global warming potential among all catalyst systems evaluated (0.0265 kg CO_2_ eq per kg IBOMA), 18–71% lower than those of H_2_SO_4_, *p*-TsOH, and betaine hydrochloride, extending the environmental advantage of TpPa–SO_3_H from mass-based waste generation to climate-change impact. These findings establish TpPa–SO_3_H as an efficient, recyclable, and environmentally favorable catalyst for bio-based specialty monomer synthesis.

## Author contributions

Q. T. Huynh: writing – original draft, formal analysis, visualization, methodology, investigation. Y. F. Lin: formal analysis, methodology, visualization. Q. T. Vo: writing – review and editing, formal analysis, methodology. K. L. Chang: conceptualization, supervision, resources, methodology, writing-review and editing. All authors have read and agreed to the published version of the manuscript.

## Conflicts of interest

The authors declare no competing interests.

## Data Availability

The data supporting the findings of this study are available within the article.
